# General practitioners' satisfaction with and attitudes to out-of-hours services

**DOI:** 10.1186/1472-6963-5-27

**Published:** 2005-03-31

**Authors:** Caro JT van Uden, Fred HM Nieman, Gemma BWE Voss, Geertjan Wesseling, Ron AG Winkens, Harry FJM Crebolder

**Affiliations:** 1 Department of Integrated Care, Research Institute Caphri, University Hospital Maastricht, P.O. Box 5800, 6202 AZ Maastricht, the Netherlands; 2 Department of General Practice, Research Institute Caphri, Maastricht University, P.O. Box 616, 6200 MD Maastricht, the Netherlands; 3 Department of Clinical Epidemiology and Medical Technology Assessment, University Hospital Maastricht, P.O. Box 5800, 6202 AZ Maastricht, the Netherlands; 4 Department of Respiratory Diseases, Research Institute Caphri, University Hospital Maastricht, P.O. Box 5800, 6202 AZ Maastricht, the Netherlands

## Abstract

**Background:**

In recent years, Dutch general practitioner (GP) out-of-hours service has been reorganised into large-scale GP cooperatives. Until now little is known about GPs' experiences with working at these cooperatives for out-of-hours care. The purpose of this study is to gain insight into GPs' satisfaction with working at GP cooperatives for out-of-hours care in separated and integrated cooperatives.

**Methods:**

A GP cooperative separate from the hospital Accident and Emergency (A&E) department, and a GP cooperative integrated within the A&E department of another hospital. Both cooperatives are situated in adjacent geographic regions in the South of the Netherlands. One hundred GPs were interviewed by telephone; fifty GPs working at the separated GP cooperative and fifty GPs from the integrated GP cooperative. Opinions on different aspects of GP cooperatives for out-of-hours care were measured, and regression analysis was performed to investigate if these could be related to GP satisfaction with out-of-hours care organisation.

**Results:**

GPs from the separated model were more satisfied with the organisation of out-of-hours care than GPs from the integrated model (70 vs. 60 on a scale score from 0 to 100; *P *= 0.020). Satisfaction about out-of-hours care organisation was related to opinions on workload, guarantee of gatekeeper function, and attitude towards out-of-hours care as being an essential part of general practice. Cooperation with medical specialists was much more appreciated at the integrated model (77 vs. 48; *P *< 0.001) versus the separated model.

**Conclusion:**

GPs in this study appear to be generally satisfied with the organisation of GP cooperatives for out-of-hours care. Furthermore, GPs working at the separated cooperative seem to be more satisfied compared to GPs working at the integrated cooperative.

## Background

During the last decade, out-of-hours care by general practitioners (GP) in the Netherlands has changed substantially. Formerly, GPs performed out-of-hours care in small locum groups in which they joined a rota system. In recent years, large GP cooperatives have been set up following British and Danish examples [1,2]. Currently, about 124 GP cooperatives are operational in the Netherlands, taking care of more than 90% of the Dutch population during out-of-hours.

In the current Dutch out-of-hours primary care, roughly two types of organisation models can be distinguished; a separated model and an integrated model. In the separated model the GP cooperative is located separate from the hospital's accident and emergency (A&E) department, indicating that there is no functional integration of out-of-hours services. In this model patients with a medical problem can choose between attending the GP cooperative or the A&E department, during out-of-hours. In the second organisation model the GP cooperative is integrated with the hospital A&E department. In this model, all patients utilising out-of-hours primary and emergency care without referral are first seen by a GP or practice nurse. It is known that a substantial number of self-referred patients at the A&E department exhibit minor injuries or non-urgent ailments that can be treated by a GP [3,4]. As a consequence, GPs of the integrated model will generally have to handle more patients than GPs at the separated model [5]. Patients with a referral or brought in by ambulance always bypass this system and will be directed to the emergency department without interference of the GP on call.

The initiative of the out-of-hours primary care reorganisation has come mainly from the medical profession itself, motivated by increased dissatisfaction with the former out-of-hours services. However, as indicated by a recently published systematic review, there is little evidence available on current GPs' satisfaction with out-of-hours services [6]. The authors of this review identified only one study with respect to GPs' satisfaction with out-of-hours care. That study showed high levels of satisfaction with cooperative based primary care services [7]. However, only a part of the GPs interviewed actually participated within this service. Other studies have reported of beneficial effects to GPs with the introduction of GP cooperatives for out-of-hours care, like improved GPs' health [8] or decreased levels of stress [9]. We have also identified one Dutch study that showed increased satisfaction after reorganising out-of-hours care from practice based to cooperative based [10]. No further information is available on GPs' satisfaction with cooperative based out-of-hours care.

There were two important reasons to conduct this study. First, because GP satisfaction has been shown to be an important contributor to quality of care [11]. GP satisfaction, besides patient satisfaction and costs, should be taken into account when evaluating out-of-hours care services. Second, during the time of the study, the integrated GP cooperative was still in its trial period and insight had to be gained in experiences and opinions of GPs working at this cooperative to support the decision whether this way of organising out-of-hours care should be continued.

The purpose of this study is to gain insight in the satisfaction of GPs with out-of-hours primary care organised in cooperatives. In addition, this study investigates potential differences in the relationship of satisfaction and other out-of-hours care related opinions between GPs working in an integrated model and GPs working in a separated model.

## Methods

This study investigates two specific elements: GPs' satisfaction with the organisation of two types out-of-hours care and the GPs' opinions related to working at either of two GP cooperatives. Two differently organised out-of-hours cooperatives are involved; a separated model and an integrated out-of-hours care model in two adjacent geographic regions in the Netherlands.

### Setting

The separated cooperative is located in the centre of the city of Heerlen, the Netherlands, about 5 km and 9 km away from the only two A&E departments in this region. This cooperative was first set up in 1999, and covered at that time a population of approximately 100,000. In 2001, more GPs joined the cooperative and the population was increased to 278,000. The number of participating GPs increased to 120. In this system patients are stimulated to make a phone call before attending the GP cooperative. This allows the GP cooperative to triage patients at urgency levels of their medical complaints in order to prioritise treatment. During out-of-hours, patients with a medical problem can choose which out-of-hours service to attend, i.e. the GP cooperative or the hospital A&E department.

The integrated GP cooperative is located in the city of Maastricht, the Netherlands, at the region's only A&E department of the University Hospital Maastricht. This cooperative was set up in January 2000. During the first one and half year, this cooperative covered only the population of the city of Maastricht (approximately 120,000). In August 2001, the surrounding area of Maastricht also joined the cooperative, increasing the coverage area to 190,000 inhabitants. In total, 83 GPs participate in the integrated GP cooperative. At this GP cooperative patients are allowed to attend the cooperative without an appointment, although it is preferred that they make a phone call first. All patients attending the integrated out-of-hours care facility without referral are first seen by a GP, who refers, if necessary, the patient to the A&E department.

At both GP cooperatives, telephone triage is performed by doctor's assistants who are supported by guidelines and protocols, and are supervised by a GP. GPs of these cooperatives perform telephone consultations, consultations at the cooperative, and home visits. Regarding home visits a chauffeured care is at their disposal. Both regions comprise rural as well as urban areas.

### Development of the questionnaire

Topics relevant to out-of-hours primary care were identified in interviews with three GPs participating in the two GP cooperatives under study. We have developed a set of items to enable us to measure and test multi-item scales. The items are related to relevant themes with respect to working at a GP cooperative. In total the questionnaire consisted of 86 items. (Some items are excluded from the analysis because they are only of local interest.) We investigated opinions on: overall satisfaction with the GP cooperative for out-of-hours, reorganisation of out-of-hours care, perceived workload, out-of-hours care as being an essential part of primary care, anonymity of care, gatekeeper function, availability of patient dossiers, cooperation with medical specialists during out-of-hours, and safety. We used a Likert five point scale (strongly agree, agree, neutral, disagree, strongly disagree) to record responses.

### Sample

In both GP cooperatives (separated and integrated) a random sample of 50 GPs was taken. In case one of these GPs was not able or refused to participate, we had a substitution list of 25 GPs for each cooperative. This list was a random sample of the remaining GPs who were not selected by the first sampling.

The questionnaire was administered by telephone to ensure high response rates. Two research assistants administered the questionnaire and received instructions, prior to the study, by FN. The study was conducted from November 2001 to February 2002.

### Statistics

Beforehand, the 86 items of the questionnaire were divided into four blocks. These blocks represented 'satisfaction with out-of-hours care organisation', 'perceptions and subjective evaluations on working conditions in the present organisation', 'opinions and beliefs on professional philosophy', and 'evaluation of the cooperation with medical specialists at the local hospital'. The most important block concerns the one in which satisfaction with out-of-hours care organisation was measured: this was operationalised with 12 items. Principal component analysis with oblimin rotation was performed on the items of this block and after removal of items with weak factor loadings (lower than +0.60 or -0.60) and/or ambiguously loading items (on more than one factor) two factors remained in analysis. Oblimin rotation can be clarified as an oblique axis-rotation technique in finding the proper factor solution within data. By using this method we wished to come to a more 'natural' solution. With oblimin rotation no orthogonal assumptions are made to the correlation(s) between factors. In this study we strove for unidimensionality as a prerequisite for independently measured scales. Four items measured satisfaction with the current cooperative (scale 1: response variable) and three items measured satisfaction with the state of affairs before the cooperative was set up (out-of-hours care in a rota system). Next, per intended scale the test stability of each factor was measured by Cronbach's alpha, and again items could be removed from this scale, if this did increase the value of the alpha coefficient. In constructing the scale "overall satisfaction with the GP cooperative" one of the four items was removed following inspection of the Cronbach's alpha, while all four had high loadings on the same factor in the principal component analysis. The item was left out of table 2 and concerned the notion that the current organisation should be restructured. Alpha went up from 0.852 on four items to 0.893 on three items. In constructing the other scales, items which were loading unambiguously high in the principal component analysis were all included after being tested on reliability by Cronbach's alpha.

**Table 2:** GP questionnaire. Description of scales and items. (Original items are in Dutch*)

**BLOCK 1. Satisfaction with out-of-hours care organisation**
**Scale 1**. Overall satisfaction with GP cooperative (Cronbach's α = 0.90; mean (SD) = 65.0 (21.6))
I am very satisfied about the functioning and the structure of the GP cooperative (+)
I am pleased with the current GP cooperative (+)
I am very satisfied with the current organisation of out-of-hours primary care (+)

**Scale 2**. Current out-of-hours care is better organised than formerly (Cronbach's α = 0.92; mean (SD) = 89.8 (19.5))
Out-of-hours care was better organised before the establishment of GP cooperatives (+)
I prefer the former organisation of out-of-hours primary care (-)
The current organisation of out-of-hours care is not an improvement compared to the former organisation (-)

**BLOCK 2. Perceptions and subjective evaluations on working conditions in the present organisation**
**Scale 3**. Experienced a high workload (Cronbach's α = 0.87; mean (SD) = 65.5 (18.4))
The workload at the GP cooperative is too high (+)
Out-of-hours care during daytime on Saturday and Sunday is very aggravating (+)
Usually, out-of-hours service is much too aggravating (+)
I do not experience such a high workload at the GP cooperative (-)
Performing out-of-hours care is absolutely not aggravating (-)
Out-of-hours care during daytime in the weekends is not too high (-)

**Scale 4**. One feels safe at the cooperative (Cronbach's α = 0.87; mean (SD) = 76.9 (18.7))
Sometimes, I feel unsafe at the GP cooperative during out-of-hours (-)
During my shifts at the GP cooperative, I never feel unsafe (+)
Regularly, I feel unsafe at the GP cooperative during out-of-hours (-)

**Scale 5**. One feels safe during home visits (Cronbach's α = 0.84; mean (SD) = 76.0 (18.4))
Regularly, I feel unsafe when performing home visits during out-of-hours (-)
Sometimes, I feel unsafe when performing home visits during out-of-hours (-)
Usually, I feel safe when performing home visits during out-of-hours (+)

**BLOCK 3. Opinions and beliefs on professional philosophy**
**Scale 6**. Out-of-hours care is an essential part of primary care (Cronbach's α = 0.97; mean (SD) = 60.3 (31.7))
These days, out-of-hours care should no longer be an essential part of primary care (-)
Out-of-hours care is definitely an essential part of primary care (+)
Out-of-hours care should always be a part of general practice (+)
There is no place anymore for out-of-hours care in general practice (-)

**Scale 7**. Anonymity of care is a problem (Cronbach's α = 0.90; mean (SD) = 32.9 (21.8))
Because the GP of the cooperative and the patient are not familiar with each other, there is a risk for inadequate treatment (+)
Because of anonymity of care there is a risk that diagnostics and treatment are not adequately adjusted for the patient's needs (+)
One of the big disadvantages of the GP cooperative is the anonymity of care, because the GP is not familiar with the patient (+)
Because the GP of the cooperative and the patient are not familiar with each other, there is a risk for inadequacy of care (+)

**Scale 8**. Gatekeeper function is well guaranteed (Cronbach's α = 0.74; mean (SD) = 64.5 (16.8))
I think the GP gatekeeper function at the GP cooperative is well guaranteed (+)
I am afraid that the GP gatekeeper function during out-of-hours will disappear (-)
I believe that in the near future the GP gatekeeper function will be put under too much pressure (-)
I think the GP gatekeeper function at the GP cooperative is well protected (+)

**Scale 9**. Availability of patient dossiers is important (Cronbach's α = 0.93; mean (SD) = 57.4 (24.1))
As far as I am concerned, I do not need the availability of my colleagues' patient dossiers (-)
I think it is a serious problem that my colleagues' patient dossiers are not at my disposal (+)
The availability of patient dossiers of the other participating GPs during out-of-hours is absolutely unnecessary (-)
During out-of-hours I am hindered in my practice, because of lack of information about my colleagues' patients (+)

**BLOCK 4. Evaluation of the cooperation with medical specialists at the local hospital**
**Scale 10**. Cooperation with medical specialists is good (Cronbach's α = 0.94; mean (SD) = 62.7 (23.0))
Sometimes, the cooperation between GPs and medical specialists is not so good (-)
I think that during out-of-hours the understanding between GPs and medical specialists is sometimes pretty bad (-)
I think that during out-of-hours the cooperation between GPs and medical specialists is always fine (+)
Generally, the cooperation between myself and the medical specialists of the hospital is good (+)

* The provisional translation into English is meant to inform the reader of the content of the scales and cannot be seen as a definite one.

Scale constructions were performed under specific rules for missing item data: in summating to a total for each case, scores had to be valid on at least half of the items, if the number of items was even, and on at least half of the items plus one half, if the number of items was uneven. Otherwise, scale scores were set to 'missing'. Finally, a transformation of the total scale score to a 0–100 score was made [12]. After that, the remaining three blocks were analysed in a similar way. In total this procedure produced ten scales.

The relationship between individual scales and overall satisfaction (scale 1) was analysed using multiple regression analysis. Upon finding the final 'direct effects' model for both regions of interest we have tested all possible interactions of pairs of significant predictors for statistical significance by the forward selection technique. Next to this, interactions were computed by multiplying predictors significant within the 'direct effects' model by the dummy 0–1 (Heerlen or Maastricht GP cooperative) coding of region. In case of missing data, listwise deletion of missing cases was applied. To test differences between GPs from either two GP cooperatives we performed independent Student's t-tests per scale. In case of non-normality, which was assessed visually by histogram analysis and by the Kolmogorov-Smirnov test, a Mann-Whitney test was used. A *P*-level of less than 0.05, was considered to be statistically significant. All data were analysed using SPSS-pc, version 10.0.5.

## Results

In total 100 GPs participated; 50 GPs per each cooperative. One respondent of the Maastricht GP cooperative (integrated model) refused to participate and was substituted by a GP from the reserve list. The mean duration of the interviews was 22 (± 6.6) minutes. The characteristics of the respondents of both models do not differ statistically (Table 1).

**Table 1:** Characteristics of respondents.

		GP cooperative Heerlen (n = 50)	GP cooperative Maastricht (n = 50)
Age		48.0 ± 7.5	47.3 ± 6.6
Gender	Male	42	43
	Female	8	7
Employed	Part-time	11	16
	Fulltime	39	34
Size of practice (GPs)	Mean (range)	2.5 (1 – 6)	2.0 (1 – 7)
Participation in GP			
cooperative	Fully	37	37
	Partly	13	13

We tested ten scales related to aspects of current out-of-hours primary care (Table 3.). Internal reliability of these scales was considered appropriate; Cronbach's alpha's ranging from 0.74 to 0.97. An overview of all scales and related items is presented in Table 2.).

**Table 3:** Scales scores for GPs opinions on different aspects of out-of-hours primary care.

	Separated Model	Integrated Model	
	(Heerlen)	(Maastricht)	

	Scale score	Scale score	Significance

Scale	Mean (95% CI)	Mean (95% CI)	

Overall satisfaction	70.0 (64.0 – 76.0)	60.0 (54.0 – 66.0)	0.020
Current out-of-hours care is better organised than formerly^b,†^	90.1 (84.7 – 95.5)	89.5 (83.7 – 95.3)	0.569**
Experience a high workload^b^	63.4 (58.1 – 68.8)	67.6 (62.5 – 72.7)	0.256
One feels safe at the cooperative^b^	76.5 (71.1 – 81.9)	77.3 (72.0 – 82.7)	0.825
One feels safe during home visits^b,‡^	74.8 (68.7 – 81.0)	77.2 (73.1 – 81.4)	0.532
Out-of-hours care is an essential part of primary care^b^	52.9 (43.7 – 62.1)	67.8 (59.4 – 76.1)	0.018
Anonymity of care is a problem^b^	31.8 (25.1 – 38.4)	34.0 (28.3 – 39.7)	0.609
Gatekeeper function is well guaranteed^b,†,‡^	66.8 (62.2 – 71.2)	62.2 (57.1 – 67.3)	0.180
Availability of patient dossiers is important^b^	55.4 (48.4 – 62.4)	59.3 (52.6 – 66.0)	0.422
Cooperation with specialists is good^b,†^	48.5 (42.1 – 54.8)	76.6 (72.9 – 80.4)	<0.001

* Scale score ranges from 0 to 100 points

^b ^100 points represents strong agreement

^† ^One case missing at the separated model, ^‡ ^one case missing at the integrated model

** Because of non-normal distribution, the Mann-Whitney test was used

GPs' overall satisfaction score with the current organisation of out-of-hours care was 65 points (95%CI: 60.7 – 69.3) on a scale from 0 (absolutely not satisfied) to 100 (highly satisfied). However, GPs from the separated model were more satisfied compared to their colleagues of the integrated model (scale score 70.0 vs. 60.0; *P *= 0.020). GPs from both cooperatives reported that the new organisation of out-of-hours primary care is better compared to the former practice-based out-of-hours care (mean scale score 89.8). Most GPs experience a high workload (mean scale score 65.5). A minority of all interviewed GPs think that the anonymity of patient care – many patients are not known to the GP because care is organised on large-scale – endangers adequacy of out-of-hours primary care (mean scale score 32.9). Furthermore, a small majority feels that the patient's medical file should be available at the cooperative (mean scale score 57.4). In both cooperative models (integrated and separated) of out-of-hours care, GPs think that their gatekeeper's role to secondary care is sufficiently guaranteed (mean scale score 64.0). Most GPs feel relatively safe at the cooperative or during out-of-hours home visits (mean scale score 76.9 and 76.0 respectively).

GPs from the separated model were neutral about out-of-hours care as being an essential part of their job as a GP, in contrast with the integrated model GPs who were more convinced that out-of-hours care is an important part of their job (scale score 52.9 vs. 67.8; *P *= 0.018). GPs at the integrated model experience a better cooperation with medical specialists during out-of-hours care (scale score 76.6 vs. 48.5; *P *< 0.001).

The regression analysis identified three scales that are significantly related to overall satisfaction (Table 4.). Effects on satisfaction for two of these scales, experienced workload and whether the GP thinks that his gatekeeper function is well guaranteed during out-of-hours, are different for both cooperatives. Experienced workload is mainly related to overall satisfaction of GPs from the integrated model. Whereas, the GP's opinion about the gatekeeper function is mainly related to overall satisfaction of GPs from the separated model. Increased experienced workload will lead to a decreased overall satisfaction, and the better the GPs valued the guarantee of their gatekeeper function during out-of-hours the higher their overall satisfaction will be.

**Table 4:** Regression analysis with overall satisfaction with the organisation of out-of-hours care as dependent variable (0 = not satisfied, 100 = very satisfied) (n = 98; R^2 ^= 0.36).

	Unstandardised coefficients		Standardised coefficient		
Scales	B	SD	Beta	t	Significance

Constant	106.352	22.154		4.801	
Age	-0.034	0.262	-0.012	-0.130	0.897
Gender ^b^	-2.188	5.228	-0.037	-0.418	0.677
Cooperative ^a^	-75.980	26.073	-1.840	-2.914	(0.005)
Gatekeeper function	0.101	0.149	0.081	0.677	(0.500)
Out-of-hours care is an essential part of primary care	-0.140	0.063	-0.212	-2.216	0.029 ^c^
Experienced workload	-0.597	0.152	-0.518	-3.915	(< 0.001)
Gatekeeper * cooperative	0.737	0.247	1.253	2.980	0.004^c^
Workload * cooperative	0.487	0.219	0.799	2.225	0.029^c^

^a ^Cooperative: Maastricht = 0; Heerlen = 1; ^b ^gender: male = 0, female = 1 ^c ^Only effects that are interpretable

Gatekeeper function (0 = not guaranteed, 100 = highly guaranteed)

Out-of-hours essential part (0 = not essential, 100 = highly essential)

Experienced workload (0 = very low, 100 = very high)

The third scale that is significantly related to overall satisfaction is the GP's opinion about out-of-hours care as being an essential part of his task as a GP. GPs who indicated that they believed that out-of-hours care was an essential part of their job as a primary care physician had a lower overall satisfaction with respect to current out-of-hours care. Neither gender nor age was significantly related to overall satisfaction. The regression model explained 36% of the variation in overall satisfaction.

Subgroup regression analysis for GPs of the integrated and separated model separately (see Table 5), showed that for the GPs of the integrated model workload was the main factor that influenced overall satisfaction (variance explained: 34%). With respect to the GPs of the separated model, the guarantee of the gatekeeper function was of great importance to the overall satisfaction (variance explained: 35%).

**Table 5:** Regression analysis results on overall satisfaction with the organisation of out-of-hours care as dependent variable for both GP cooperatives separately (0 = not satisfied, 100 = very satisfied).

	Unstandardised coefficients		Standardised coefficient		
	B	SD	Beta	t	Significance

**Separate GP cooperative (R^2 ^= 0.35) n = 49**					

Constant	21.823	20.437		1.068	0.291
Age	-0.122	0.366	-0.043	-0.333	0.741
Gender ^a^	-0.110	7.475	-0.002	-0.015	0.988
Gatekeeper function	0.956	0.201	0.697	4.755	<0.001
Out-of-hours care is an essential part of primary care	-0.182	0.098	-0.273	-1.851	0.071

**Integrated GP cooperative (R^2 ^= 0.28) n = 50**					

Constant	101.692	23.204		4.383	<0.001
Age	0.073	0.398	0.023	0.182	0.856
Gender ^a^	-9.375	7.335	-0.156	-1.278	0.208
Experienced workload	-0.648	0.144	-0.551	-4.496	<0.001

^a ^Gender: male = 0, female = 1

Gatekeeper function (0 = not guaranteed, 100 = highly guaranteed)

Out-of-hours essential part (0 = not essential, 100 = highly essential)

Experienced workload (0 = very low, 100 = very high)

Adding the scale of experienced workload to the regression equation of the Separate GP cooperative in Table 5 will make variance explained in overall satisfaction only higher by 0.007 to 0.359 (F ratio of the change = 0.50 by 1 and 43 df., p = 0.482). Adding the scales of gatekeeper function and out-of-hours care as an essential part of primary care to the regression equation of the Integrated GP cooperative will make variance explained in overall satisfaction only higher by 0.036 to 0.317 (F ratio of the change = 1.13 by 2 and 43 df., p = 0.332).

## Discussion

GPs in this study are generally satisfied with the way out-of-hours primary care is currently organised. However, GPs from the separated cooperative are more satisfied than GPs working at the integrated cooperative. Mainly three factors are related to overall satisfaction. These are: experienced workload, guarantee of the gatekeeper function, and attitude towards out-of-hours care as being an essential part in general practice.

To our best knowledge, this is the first study to investigate GPs' satisfaction with out-of-hours care as organised in separated and integrated primary care cooperatives. One British study and one Dutch study have looked into GPs satisfaction with out-of-hours care[7,10]. However, these studies solely focused on separated out-of-hours care models. The Dutch study showed that 70% of the GPs were satisfied with cooperative based out-of-hours care [10], and the British study found that 92% of the GPs were satisfied with the way out-of-hours care was arranged [7]. Since these studies used different ways to measure satisfaction it is difficult to compare them with our results.

The results of this study indicate a difference in satisfaction between GPs from the separated and integrated cooperative. A possible explanation for this difference could be the fact that the integrated cooperative has to deal with a larger number of patients compared to the separated cooperative [5]. In this study however, experienced workload of GPs from the integrated cooperative did not differ from that of the GPs of the separated one. Obviously workload is also dependent on staffing of the cooperative. We presume that the difference in satisfaction might well be explained by other factors, which we have not investigated in this study. At the time of the study the integrated model was still in its experimental phase; housing in the integrated cooperative was generally not considered to be optimal. In this phase of the experiment the waiting room was very small and quickly overcrowded. Also, the space in the doctor's offices was quite limited and contained only room for one bed and no desk. In addition, at this time also patients' and GPs' privacy were not as sufficiently guaranteed as in the separated model. These factors may have had an effect on GPs' overall satisfaction with out-of-hours services.

Three opinions were found to be significantly related to GP satisfaction with the organisation of out-of-hours care. The two opinions that weighted most heavily on satisfaction were experienced workload and gatekeeper function.

We found that the GPs' opinion on the gatekeeper function during out-of-hours was related to satisfaction with the organisation of out-of-hours care specifically for GPs of the separated GP cooperative. The fact that this opinion is not related to satisfaction with the organisation in the integrated cooperative is probably due to the fact that this is not an issue at this cooperative, because the GP's gatekeeper function is fully guaranteed; all patients entering the out-of-hours centre are screened by a GP and if necessary referred to a medical specialist. In the separated model however, the patient can still bypass the GP and attend the emergency department of the hospital without a GP's referral. GPs who feel to have too little grip on these self-referring patients appear to be less satisfied with their arrangements of out-of-hours care.

We have not been able to investigate GPs' satisfaction prior to the reorganisation from practice based out-of-hours care to cooperative based out-of-hours care, whilst GPs' dissatisfaction with practice based out-of-hours care was one of the important reasons why primary care in the Netherlands was reorganised. Nevertheless, this study shows that GPs feel that current out-of-hours primary care is better organised compared to former practice-based out-of-hours care. These results are in line with previous research [10]. However, this is not surprising considering the effort that has gone into reorganising the out-of-hours services and the prior dissatisfaction. All those who were in favour of the change of the out-of-hours system will obviously be satisfied with the fact that out-of-hours care has been reorganised, and feel that the new system is better than formerly.

A distinct feature of the integrated model is the close cooperation between primary and hospital emergency care. This offers possibilities to improve communication and to exchange expertise. This is reflected by the high satisfaction score of GPs from the integrated model with the cooperation with the medical specialists of the hospital. Because GPs and medical specialists now work at the same site, it is easier to consult each other. Furthermore, GPs who have referred a patient to one of the medical specialists have access to feedback, i.e. they can check on the patient a few minutes later to see if they were right in their diagnosis. Nevertheless, region-specific differences may also have accounted for this difference, because in the region with the integrated cooperative there is a longer tradition in cooperation between primary and secondary care.

We investigated GPs' opinions on working at two contrasting models of out-of-hours primary care, i.e. a separated and an integrated GP cooperative, in order to gain insight in GPs' preferences for either one of these models. Until November 2001, the Maastricht GP cooperative for out-of-hours care was the only cooperative in the Netherlands that was integrated with a hospital A&E department. Consequently, the Maastricht out-of-hours care organisation was the only service at the time of the study that could be used as an example of integrated out-of-hours care.

There are limitations to generalise the results of the study to other regions. First, results of the study reflect the opinions of GPs at only two cooperatives in the South of the Netherlands. Second, the integrated GP cooperative was still in its trial phase and may therefore have not been a good representative of a well-established GP cooperative. Nevertheless, this is the first study to address GP satisfaction with an integrated GP cooperative and may therefore, despite the limited generalisability, give some indication of relevant aspects of integrated out-of-hours care for further research and care development.

Currently, three regions in the Netherlands are working according to an integrated out-of-hours care system. However, at the moment GPs in other regions consider adopting this organisational structure. Furthermore, the current Dutch minister of health care has stated to be in favour of an intensive collaboration between primary and emergency care, for this will probably reduce costs [13]. The results of this study can support the current discussion in the Netherlands on the organisation of out-of-hours primary care.

Future research should focus on the economic efficiency of both models and patient preference with respect to the organisation of out-of-hours primary care, because these are important features to take into account when developing out-of-hours care.

## Conclusion

GPs in this study appear to be generally satisfied with the organisation of GP cooperatives for out-of-hours care. Furthermore, GPs working at the separated cooperative seem to be more satisfied compared to GPs working at the integrated cooperative.

## Competing interests

The author(s) declare that they have no competing interests.

## Authors' contributions

CvU, FN, GV, RW, GW, and HC participated in the design of the study. CvU, FN and GV developed the questionnaire. CvU and FN performed statistical analysis. CvU drafted the manuscript. FN, GV, RW, GW, and HC provided critical edits to this manuscript. HC supervised the study. All authors have read and approved the final manuscript.

## Pre-publication history

The pre-publication history for this paper can be accessed here:


